# Magnitude of Turnover Intention and Associated Factors among Health Professionals Working in Public Health Institutions of North Shoa Zone, Amhara Region, Ethiopia

**DOI:** 10.1155/2018/3165379

**Published:** 2018-12-23

**Authors:** Aster Ferede, Getiye Dejenu Kibret, Yihenew Million, Muluye Molla Simeneh, Yihalem Abebe Belay, Damen Hailemariam

**Affiliations:** ^1^Department of Public Health, College of Health Sciences, Debre Markos University, Debre Markos, Ethiopia; ^2^Department of Medical Microbiology, School of Biomedical and Laboratory Sciences, College of Medicine and Health Sciences, University of Gondar, Gondar, Ethiopia; ^3^Department of Reproductive Health and Health Service Management, School of Public Health, College of Health Sciences, Addis Ababa University, Addis Ababa, Ethiopia

## Abstract

**Background:**

Health workers are one of the most important building blocks of the health system. High turnover rate contribute to the shortfalls and unbalanced distribution of health personnel in the health workforce. Turnover intention is the strongest predictor of actual turnover.

**Objective:**

To assess the magnitude of turnover intention and associated factors among health professionals working in public health institutions of North Shoa Zone, Amhara region, Ethiopia.

**Methods:**

A health facility based cross-sectional study was conducted from March to April 2016, on 612 health professionals working in public health institutions of North Shoa Zone, Amhara region, Ethiopia, using a multistage stratified sampling technique. Data were collected using a pretested self-administered structured questionnaire. The data were entered using Epidata version 3.1 and analyzed using SPSS version 22 software. Descriptive statistics were conducted to summarize the sample characteristics. Backward stepwise logistic regression model was fitted and AOR with 95% CI was calculated to identify the associated factors. P-value <0.05 was taken as a cut-off point for statistical significance. Ethical issues were addressed.

**Results:**

Among the 568 health professionals who responded to the questionnaire, 348 (61.3%) of them reported to have the intention to leave their current workplaces. The results showed that being a male [AOR = 1.496 (95% CI: 1.016, 2.204)], medical doctor [AOR = 0.318 (95% CI: 0.122, 0.824)], unsatisfied with the work nature [AOR = 1.822 (95% CI: 1.206, 2.753)], unsatisfied with the incentives [AOR = 1.668 (95% CI: 1.105, 2.517)], and unsatisfied with the supervision [AOR = 1.916 (95% CI: 1.274, 2.881)] and having low normative commitment [AOR = 2.176 (95% CI: 1.482, 3.196)] were significantly associated with turnover intention of the health professionals.

**Conclusions:**

The magnitude of turnover intention was high among health professionals working in public health institutions of North Shoa Zone. Health service managers and policymakers should develop evidence based retention strategies considering the determinants of health professionals' intention to leave.

## 1. Background

Health workers are one of the most important building blocks of the health system without which global and national efforts to achieve the health related goals cannot be reached to the set targets [1]. Professional mix and adequate number of health care providers available in a country play a key role in the efforts to fulfill the various health care needs of the society. Health workforce shortage and retention problems have been the major challenges in the health sector [2]. Globally, it was estimated there to be a deficit of about 7.2 million health professionals which could increase twofold in the next few decades [3]. The worker shortfall and global disease burden is more prominent in Africa, particularly sub-Saharan countries, having the least health worker to population ratio, which is not sufficient to meet the needs of the rapidly growing population [4]. Ethiopia, despite having the highest number of health workers from sub-Saharan Africa, has been suffering from human resource for health crisis [5]. The national estimated density of health workers (0.84 per 1000 population) and the distribution of physicians (0.03 per 1000 population) showed the lowest thresholds with less likely feasibility of achieving the minimum possible threshold of worker density necessary to attain the set health system goals [6].

Turnover, which is usually expressed in terms of the percentage of staff of a particular job who has left the organization [7], is one of the main reasons for the shortfalls and unbalanced distribution of health personnel [2]. It can be either voluntary or involuntary [8, 9]. Existing studies confirmed that actual turnover can be best predicted by studying the employee's intent to leave their current organization [10, 11].

Health workers' decision to stay or leave their posts is often influenced by a number of individual, organizational, and job related factors [12, 13]. Job satisfaction [14, 15] and its particular dimensions such as work nature [16, 17], work environment [16, 18, 19], autonomy [20], supervision [21, 22], remunerations [23–26], and peer group relationships [27, 28] are among the major predictors. Others reported that organizational commitment, which refers to the feeling of attachment to the goals and values of an organization [29] and its individual components (affective commitment, continuance commitment, and normative commitment), has been linked with the intent to leave [30–36]. Various demographic and personal characteristics were also determinants of turnover intention as evidenced in different literature [37, 38].

Staff instability in health care organizations would lead to various negative consequences, both at the individual and organizational levels. Loss of experienced employees with their valuable knowledge is followed by extensive costs imposed on the organization for communicating job vacancies, recruitment, selection, hiring, and retraining purposes. This in turn can reduce organizational performance and employee productivity. In addition, it disrupts the performance and morale of the remaining staff and increases their workload, resulting additional burdens. Moreover, the quality of health care services would be compromised leading to unfavorable patient outcomes [39, 40].

According to the data obtained from North Shoa Zone health office, a total of 206 health professionals left their working health facilities in 2014/15 fiscal year which alarmed a high turnover. Identifying the causes and signals of turnover is the first step in taking the appropriate measures to reduce turnover rates. This study aimed to assess the magnitude of turnover intention and associated factors among health professionals working in public health institutions of North Shoa Zone and it could provide insights for policymakers to develop the appropriate retention strategies and programs for attracting health professionals.

## 2. Methods

### 2.1. Study Design and Setting

A health facility based cross-sectional study was conducted from March to April 2016 among Health professionals working in public health institutions in North Shoa Zone, which is found in Amhara National Regional State. The capital city of North Shoa Zone, Debre Birhan town, is located 130 kilometers away from Addis Ababa, the capital city of Ethiopia, to the North East. According to the information obtained from North Shoa Zone health bureau, there were 25 woredas in that zone with a total health worker population of 3585 including administrative staff.

#### 2.1.1. Sample Size and Participants

The study populations were all randomly selected health workers deployed to work in the selected woredas who served for six months or greater in health institutions. Health workers who were ill, on maternity leave, annual leave, and study leave, were excluded from the study. The sample size was calculated using a single population proportion formula by taking the proportion (p) of turnover intention of health professionals to be 59.4% [30]. The assumptions of 95% confidence level, 5% margin of error, and a design effect of 1.5 were considered. After adjusting 10% for possible nonresponse, the final sample size was 612.

#### 2.1.2. Sampling Procedure

A multistage stratified sampling technique was used. Among the 25 woredas found in North Shoa Zone, 10 woredas were selected by simple random sampling using a lottery method. Then, they were stratified into two woredas containing both hospitals and health centers (Debrebirhan and Efrata ena Gidim) and eight woredas containing only health centers (Angolelana Tera, Baso Ena Werana, Ankober, Tarmaber, Moret Ena Jiru, Mida Weremo, Limu, and Ansaro). The total number of health professionals in these 10 woredas (1117) and the total sample size were used to proportionally allocate for each woredas based on the specific types of professions. Eligible health workers in the selected woredas were taken by simple random sampling using a lottery method.

#### 2.1.3. Study Instruments

The dependent variable was turnover intention and the independent variables included sociodemographic characteristics, factors related to job satisfaction such as work nature, work environment, incentives, supervision, autonomy, and peer group relationship and organizational commitment and its subscales (normative, affective, and continuance commitment). The data were collected by using a pretested structured self-administered questionnaire which was developed after doing a literature review.

Turnover intention was measured with three item scales (*α* = 0.878) obtained from a literature review [41–46]. These items were added up and those who scored above the mean were labeled as yes and those below the mean were labeled as no. Job satisfaction was measured with 37 items divided into six subscales such as work nature, work environment, remuneration, supervision, autonomy, and peer group relationships each having their own measuring items adopted from different literature and modified accordingly [47–50]. These items were added up and those above the mean score were labeled as satisfied while those below the mean were labeled as unsatisfied.

Organizational commitment was measured with three subscales consisting normative, affective, and continuance commitment, as obtained from different literature [42, 51–53]. Three items that measure normative commitment, four items that measure continuance commitment, and four items that measure affective commitment were added up and those above the mean value were labeled as having a high score while those below the mean value were labeled as having a low score accordingly. Response choices for each item were given with a 1-5 Likert scale, with 1 denoting strongly disagree, and 5 denoting strongly agree and negative items were reverse coded. The questionnaire was prepared in English, then, translated into Amharic and evaluated. Four supervisors and ten experienced data collectors were selected from the study area by the principal investigator. They were given training for two days on data collection techniques. The tasks of the supervisors included deploying and assisting the data collectors.

#### 2.1.4. Data Analysis

Data were entered using Epidata version 3.1 and exported to SPSS version 22.0 for analysis. After data cleaning was done, the variables were recoded and dichotomized using the mean values as a cutoff point accordingly. Descriptive statistics and bivariable analysis were done and variables with p-value<0.05 were fitted into a backward stepwise logistic regression model. Odds ratio and 95% confidence intervals were calculated. A P-value less than 0.05 was used to determine statistical significance.

#### 2.1.5. Data Quality Control

To achieve a good data quality, before commencing data collection, the questionnaire was pretested (5%) in the woredas that were not selected to ensure its validity and to standardize the study instrument locally. The data collection was supervised and followed throughout the study period by the supervisors and the principal investigator. They went to each selected woredas to check for the proper filling of the questionnaires. In addition, each questionnaire was given a code. Epi data was used to control data entry errors and data cleaning was performed using SPSS.

#### 2.1.6. Ethics Approval Statement

Ethical clearance was obtained from the institutional review board of college of health sciences, Addis Ababa University. The necessary permission to undertake the study was obtained from North Shoa Zone health bureau and respective Woreda health offices. The study participants were properly instructed and indicated that they consent to participate by signing on the written informed consent form provided.

## 3. Results

### 3.1. Sociodemographic Characteristics of the Study Participants

A total of five hundred sixty-eight respondents returned the questionnaires, making a response rate of 92.8%. The mean (±Standard Deviation) age of the health professionals was 27.86 (±5.455SD) which ranged from 20-57 years. Three hundred thirty-two (58.5%) of them were males. The majority of the respondents were single 326 (57.4%), orthodox Christian 516 (90.8%), and Amhara 520 (91.5%) regarding their marital status, religion and ethnicity, respectively. 271 (47.7%) of the health workers were nurses. The majority of them, 345 (60.5%), were working in health centers and 347 (61.1%) of them had a diploma. Of the total study participants, 190 (33.5%) of them worked in health facilities located in their home town and had established family. 167 (29.4%) of them had children for whom they must provide care. The mean (±SD) experience and monthly salary were 3.3932 (±4.32SD) years and 2755.38 (±1371.54) Ethiopian Birr (ETB) respectively. Only 12 (2.1%) of them had alternative sources of income (Table 1).

### 3.2. Turnover Intention

Out of the 568 health professionals, 348 (61.3%) of them had the intention to leave their current health facility.

### 3.3. Factors Related to Job Satisfaction

The results indicated that three hundred fifty-six (62%) of the health workers were satisfied with the work nature while 322 (56.7%) and 400 (70.4%) of them were satisfied with autonomy and peer group relationships in the health facilities, respectively. Nevertheless, the majority of the respondents, 53.9%, 53.9%, and 50.4%, were unsatisfied with the work environment, remuneration, and supervision, respectively (Table 2).

### 3.4. Factors Related to Organizational Commitment

The results revealed that considering the individual dimensions of organizational commitment, about 53% and 59% of the health workers had low normative and continuance commitment, respectively, while the majority (57.9%) of them had a high affective commitment (Table 3).

### 3.5. Factors Associated with Health Professional's Turnover Intention

Bivariable analysis was performed and taking p-value<0.05 as a cut-off point for determining significance, twelve variables showed a significant association with the outcome variable. These were sex, profession, ethnicity group, establishing family, job satisfaction facets such as work nature, work environment, incentives, supervision, and autonomy, and the components of organizational commitment such as normative, continuance, and affective commitments. Sociodemographic characteristics such as age, marital status, religion, educational status, and experience did not show a significant association with turnover intention. In addition, having children, type of health facility, having an alternative source of income, and job satisfaction with peer group relationships were not associated with the intent to leave.

### 3.6. Multivariable Binary Logistic Regression Analysis Results

The twelve covariates that were significantly associated (p-value<0.05) with turnover intention were fitted into the backward stepwise multivariable binary logistic regression model to control for confounding. Nine variables (normative commitment, continuance commitment, satisfaction with supervision, satisfaction with incentives, satisfaction with work nature, sex, family, ethnicity, and profession) appeared in the multivariable model after stepwise selection process. The final predictors of turnover intention were sex, profession, satisfaction with work nature, satisfaction with incentives, satisfaction with supervision, and having a low normative commitment (Table 4).

## 4. Discussion

Human resources are the most important assets in the health system. Developing and retaining capable and motivated health workers is crucial for advancing the quality of health care services [4]. This cross-sectional study aimed at examining the magnitude of health worker's turnover intention and the factors associated with it. This is of an important issue because turnover intention is the strongest predictor of actual turnover. An effective strategy for dealing with it is identifying the prevalence of turnover intention with its determinants and taking measures to reverse this intention thereby reducing the costs and other consequences of turnover on the organization. In this study, more than half of the respondents (61.3%) had the intention to leave their current workplaces. Various factors related to turnover intention were also examined. The findings revealed that turnover intention was significantly associated with sex, profession, satisfaction with the work nature, satisfaction with incentives, satisfaction with supervision, and normative commitment.

The magnitude of turnover intention, we found in this study, is similar to studies conducted in Jimma zone (63.7%) [54] and East Gojjam zone (59.4%) [30]. But, a study done in Gondar reported a slightly lower (52.5%) magnitude than this study [37]. This variation could be attributed to the difference in the health facility. Our study considered both hospitals and health centers whereas the study from Gondar considered health workers only in a hospital. A study done in Sidama [27] also found a slightly lower proportion (50%) than the present study, which can be explained by the difference in the health professions. The study done in Sidama included only a single discipline of profession unlike our study. Other studies conducted in Tanzania, Malawi, and South Africa revealed lower proportion (18.8%, 26.5%, and 41.4%), respectively [55]. This variation may be attributed to the presence of successful ongoing interventions to address the problem in these countries. In addition, a study done among Chinese doctors [56] and Italian nurses [57] indicated the magnitude to be 36% and 21%, respectively. This variation may be due to the presence of attractive salary and other incentives and better infrastructures in these developed countries.

With regard to sociodemographic characteristics, sex and profession were found to be the predictors of the health workers intent to leave their current organization. Our finding indicated that males were more likely to consider leaving than females, which is consistent with studies conducted in Saudi Arabia [38] and Iraq [58]. A possible explanation for this is that females may prefer to remain stable to moving out than males. Yet, in studies conducted in two different hospitals in South Africa, gender was stated as not influencing the intent to leave [26].

Our result also showed that profession was the other determinant factor in that medical doctors were less likely to have the intent to leave than other health professionals. This may be attributed to the availability of alternative sources of income for medical practitioners such as working in universities and hospitals at the same time with dual payment, working in private clinics and together with its proximity to the capital city, Addis Ababa, which offers more opportunities to work at these two different places. Our result is in line with a finding from Gondar University hospital [37]. Other socioeconomic and demographic factors such as age, marital status, educational status, and income did not play a significant role in the respondent's decision to leave out. Similar findings were found in a South African study [59].

As for the individual dimensions of job satisfaction that were linked with the intention to quit, health workers who were dissatisfied with supervisory support were more likely to leave than those who were satisfied. This finding is similar to studies done in public health centers in Jimma [54], Japan [60], and Turkey [22]. This finding can be explained in that recognition and support by supervisors and positive feedback for individual performances enhance the feeling of responsibility, honesty, and autonomy. Poor supervisory support occupied by partiality may reduce job satisfaction leading to the desire to leave the organization.

The other aspect explaining job satisfaction, remuneration, was also among the significant predictors in which health professionals who were not satisfied with the salary and benefits provided a thought of leaving than their counterparts. This result is consistent with studies conducted in China [56] and Senegal [61]. This can be explained in that health workers who perceive that the financial or nonfinancial incentives are not adequate to meet their needs may prefer leaving the organization to stay, whereas studies done in Sidama Zone [27] and Ghana [62] revealed that remuneration was not associated with the intent to leave the current workplace.

Health workers who reported that they were dissatisfied with the work nature were more likely to consider leaving than those who were satisfied, which was also supported in a study conducted in South Africa [16]. Health workers who do not like the things they do at work and luck a feeling of accomplishment may prefer to leave their workplace than stay.

The other determinant factor for turnover intention of health workers from this study was normative commitment. The health professionals with a low level of normative commitment had more likely intent to leave their current health facility than those with a high level of it. Similar finding was revealed in a study done in Jordan [63]. This finding can be explained in that health workers who do not feel they have the obligation to remain and serve their organization may reveal the desire to leave out than staying.

## 5. Conclusions

Overall, the turnover intention among health professionals working in public health institutions of North Shoa Zone is high. The mean age of the respondents indicated that the majority of the health workers are young and with less work experience. Most of the health workers were satisfied with the work nature, autonomy, and peer group relationships, but the work environment, remuneration mechanisms, and supervision were reported dissatisfying by most of the health professionals. Sex, profession, and job satisfaction with the work nature, job satisfaction with incentives, job satisfaction with supervision, and normative commitment are the final predictors for the intent to leave the health care institutions. Health care policymakers, health service managers, supervisors, and other stakeholders need to develop and implement retention strategies that aim at creating an attractive work nature, remuneration mechanisms, and supervisory support and make efforts to develop a sense of obligation in the health workers.

## Figures and Tables

**Table 1:** Sociodemographic characteristics of health professionals working in public health institutions of North Shoa Zone, Amhara Region, Ethiopia, 2016 (n = 568).

Variables		Frequency	Percentage (%)
Age (years)	<=25	217	38.2
	26-30	247	43.5
	31-35	51	9.0
	>35	53	9.3
Sex	Male	332	58.5
	Female	236	41.5
Marital status	Single	326	57.4
	Married	207	36.4
	Divorced	12	2.1
	Separated	23	4.0
Religion	Orthodox Christian	516	90.8
	Muslim	22	3.9
	Protestant	24	4.2
	Others	6	1.1
Ethnicity	Amhara	520	91.5
	Others	48	8.5
Profession	Nurse	271	47.7
	health officer	57	10.0
	medical doctor	25	4.4
	others*∗*	215	37.9
Health facility	Health center	345	60.7
	Hospital	223	39.3
Educational status	Diploma	347	61.1
	degree and above	221	38.9
Established family	Yes	190	33.5
	No	378	66.5
Had children	Yes	167	29.4
	No	401	70.6
Experience in years	<=2	308	54.2
	2-4	137	24.1
	>=4	123	21.7
Salary (ETB)	1000-2000	212	37.3
	2100-3000	205	36.1
	3100-4000	75	13.2
	>4000	76	13.4
Home town	Yes	190	33.5
	No	378	66.5
Had alternative source of income	Yes	12	2.1
	No	556	97.9

*∗*others: pharmacy, laboratory, midwifery, environmental health, and anesthesia professionals.

**Table 2:** Job satisfaction factors among health professionals working in public health institutions of North Shoa Zone, Amhara Region, Ethiopia, 2016 (n = 568).

Variable		Frequency	Percent (%)
Job satisfaction			
Work nature	Unsatisfied	212	37.3
	Satisfied	356	62.7
Work environment	Unsatisfied	306	53.9
	Satisfied	262	46.1
Remuneration	Unsatisfied	306	53.9
	Satisfied	262	46.1
Supervision	Unsatisfied	286	50.4
	Satisfied	282	49.6
Autonomy	Unsatisfied	246	43.3
	Satisfied	322	56.7
Peer group relations	Unsatisfied	168	29.6
	Satisfied	400	70.4

**Table 3:** Factors related to organizational commitment among health professionals working in public health institutions of North Shoa Zone, Amhara Region, Ethiopia, 2016 (n = 568).

Variable		Frequency	Percent (%)
Organizational commitment			
Affective commitment	Low	239	42.1
	High	329	57.9
Continuance commitment	Low	339	59.7
	High	229	40.3
Normative commitment	Low	304	53.5
	High	264	46.5

**Table 4:** Bivariable and multivariable logistic regression analysis of factors associated with turnover intention of health professionals in North Shoa zone, 2016 (n = 568).

Variables		Turn over intention		Odds Ratio		P-value
		Yes	No	COR with 95% CI	AOR with 95% CI	
Sex						
	Male	219	113	1.61 (1.14, 2.26)	**1.49 (1.02, 2.20)**	**0.04**
	Female	129	107	1.00	1.00	
Established family						
	Yes	104	86	1.00	1.00	
	No	244	134	1.51 (1.05, 2.14)	1.5 (0.98-2.21)	0.06
Ethnicity						
	Amhara	312	208	1.00	1.00	
	Others	36	12	2.0 (1.01, 3.93)	2.01 (0.97-4.2)	0.06
Profession						0.02
	Nurse	167	104	1.05 (0.72, 1.51)	1.03 (0.68, 1.54)	
	Health officer	42	15	1.8 (0.95, 3.50)	1.7 (0.85, 3.53 )	
	Medical doctor	9	16	0.4 (0.15, 0.87)	**0.32 (0.12, 0.82)**	**0.01**
	Others	130	85	1.00	1.00	
Work nature						
	Unsatisfied	156	56	2.4 (1.64, 3.44)	**1.8 (1.21, 2.75)**	**0.004**
	Satisfied	192	164	1.00	1.00	
Remuneration						
	Unsatisfied	223	83	2.9 (2.07, 4.17)	**1.7 (1.10, 2.51)**	**0.01**
	Satisfied	125	137	1.00	1.00	
Supervision						
	Unsatisfied	210	76	2.9 (2.03, 4.09)	**1.9 (1.27, 2.88)**	**0.002**
	Satisfied	138	144	1.00	1.00	
Normative commitment						
	Low	223	81	3.06 (2.15, 4.35)	**2.18 (1.48, 3.19)**	**<0.001**
	High	125	139	1.00	1.00	
Continuance commitment						
	Low	231	108	2.05 (1.44, 2.89)	1.5 (0.99-2.2)	0.05
	High	117	112	1.00		

## Data Availability

All the data supporting the study findings are within the manuscript. Additional detailed information and raw data are available from the corresponding author on reasonable request.

## Abbreviations

AOR: Adjusted odds ratio
CI: Confidence interval
COR: Crude odds ratio
ETB: Ethiopian Birr
SD: Standard deviation
SPSS: Statistical package for social sciences.

## Supplementary Materials

Supplementary Materials English version questionnaire.
