# Supplementary material for: Magnitude of Turnover Intention and Associated Factors among Health Professionals Working in Public Health Institutions of North Shoa Zone, Amhara Region, Ethiopia
Source: Biomed Res Int. 2018 Dec 23;2018:3165379. doi: 10.1155/2018/3165379 (PMC6323441; doi:10.1155/2018/3165379)
Supplement: Supplementary Materials — English version questionnaire. [file 3165379.f1.docx]

Appendix I: Participants information sheet

Good morning/afternoon. My name is __________________________________________

I am a graduate student in the school of public health at Addis Ababa University. I kindly ask you to help us in the study we are conducting, turnover intention and associated factors. The purpose of the study is to gather information on the magnitude of turnover intention and associated factors among health professionals in North Shoa zone.

I would like your permission to discuss with you about your perceptions, ideas, and experiences related to the intention to leave. No one will charge you for your participation or give you any money, whether or not you agree to fill the questionnaire. Your participation is voluntary and you don’t have to answer any particular questions if you prefer not to respond or you may end the discussion at any time. Everything you say will be kept private and confidential. I want to assure you that your participation in the study will not affect you and your institution.

If you have any questions you can ask any time. Your name will not be used in any report, but your ideas and suggestions will help us to attain our objective. Please feel free to answer exactly as you feel. If you are clear with the information provided and agree to participate please sign on the consent form attached.

Are you willing to participate in this study?

1. [ ] Yes 2. [ ] No

**Thank you for your time**

**Appendix II:** Informed consent form

I the undersigned individual have been informed about the purpose of this particular research project. I have been informed that I am going to respond to this question by answering what I know concerning the issue. I have been informed that the information I give will be used only for the purpose of this study and my identity as well as the information I give will be treated confidentially. I have also been informed that I can refuse to participate in the study or not to respond to the questions if I am not interested. Furthermore, I have been informed that I can stop responding to the questions at any time in the process. Based on the above information I agree to participate in this research voluntarily.

Signature_____________________________

Date_________________________________

If there are things that require clarification, please don’t hesitate to ask the interviewer or the principal investigator for clarification.

**Address of the principal investigator**

Aster Ferede

Addis Ababa University, school of public health

Mobile: 0910616587

**Advisor-** Professor Damen Hailemariam

Addis Ababa University, school of public health

**Appendix III: Questionnaire**

**Addis Ababa University**

**College of Health sciences**

**School of public health**

**Dear respondents;**

This questionnaire is designed to gather information on the magnitude of turnover intention and associated factors among health professionals working in public health institutions of North Zone, Amhara region. The purpose of the study is exclusively for academic requirement. I assure you that, all your responses will be kept in absolute confidentiality and you will not be held responsible for the research outcome. Therefore, your genuine, frank and timely responses are quite vital to determine the success of this study. So, I kindly request your contribution in filling the questionnaire honestly and responsibly.

**NB.**

- No need of writing your name.
- Encircle the letter of your choice and put the [🗸] mark in the box provided.
- Please respond as accurately as possible and at your earliest possible time.

**Thank you for your cooperation!**

**Part 1: Socio-demographic characteristics of the respondents**

Instruction: Please circle the number in front of the option you choose on the right side of the table.

| **No** | **Variable** | **Response** | **skip** |
| --- | --- | --- | --- |
| 1 | Age | ______________ |  |
| 2 | Sex | 1. Male 2. Female |  |
| 3 | Marital status | 1. Single 2. Married 3. Divorced 4. Widowed |  |
| 4 | Religion | 1. Orthodox Christian 2. Muslim 3. Protestant 4. Other |  |
| 5 | Ethnicity | 1. Amhara 2. Oromo 3. Tigrea 4. Others |  |
| 6 | Have you established family in the place you are living currently? | 1. yes 2. no |  |
| 7 | Do you have children for whom you must provide care? | 1. yes 2. no |  |
| 8 | What is your profession? | 1. Nurse 2. Health officer 3. Medical doctor 4. Pharmacy professional 5. Laboratory professional 6. Midwifery 7. Other |  |
| 9 | What is your educational status? | 1. Diploma 2. 1^st^ degree 3. 2^nd^ degree 4. Other |  |
| 10 | What is the Type of health facility where you work in currently? | 1. Health center 2. Hospital |  |
| 11 | Is the health facility where you work in located at your home town? | 1. Yes 2. No |  |
| 12 | How long have you been working in the current health facility (in years)? | ____________________ |  |
| 13 | How much is your current monthly salary? | ____________________ETB |  |
| 14 | Do you have any alternative source of income out of your current organization? | 1. yes(specify if yes)_____________ 2. no |  |

**Part 2: Intention measuring items**

Instruction: These are statements about health professional’s ‟ intent to leave the organization, and each statement has five alternatives with five-point scale. Read each item carefully and tick

| **No** | **Turnover intention measuring items** | **Scale** | | | | |
| --- | --- | --- | --- | --- | --- | --- |
|  |  | **Strongly disagree** | **Disagree** | **neutral** | **agree** | **Strongly agree** |
| 1 | I often think of leaving this organization |  |  |  |  |  |
| 2 | I am actively looking for a job in another organizations |  |  |  |  |  |
| 3 | I plan to leave my organization as soon as possible |  |  |  |  |  |

**Part 3: Job satisfaction factors questionnaire**

Instruction: These are statements about job satisfaction factors affecting health professional’s turnover intention and each statement has five alternatives with five-point scale. Read each item carefully and tick:

| **No** | **Statements of work nature** | **Scale** | | | | | | | | | | |  |
| --- | --- | --- | --- | --- | --- | --- | --- | --- | --- | --- | --- | --- | --- |
|  |  | **Strongly disagree** | **disagree** | | **neutral** | | **agree** | | **Strongly agree** | | | |  |
| 1 | I like doing the things I do at work |  |  | |  | |  | |  | | | |  |
| 2 | My work gives me a feeling of personal accomplishment |  |  | |  | |  | |  | | | |  |
| 3 | I feel a sense of pride in doing my job |  |  | |  | |  | |  | | | |  |
| 4 | My work allows me to use my skills and abilities optimally |  |  | |  | |  | |  | | | |  |
|  | **Statements of work environment** | **Scale** |  | | | | | | | | |  |  |
|  |  | **Strongly disagree** | **disagree** | | **neutral** | | **agree** | | **Strongly agree** | | | |  |
| 1 | The workload is manageable |  |  | |  | |  | |  | | | |  |
| 2 | I have sufficient work space to do my job |  |  | |  | |  | |  | | | |  |
| 3 | Staffing levels at my work place are adequate |  |  | |  | |  | |  | | | |  |
| 4 | I have the equipment I need to do my job properly |  |  | |  | |  | |  | | | |  |
| 5 | There are enough drug supplies for me to do my job well |  |  | |  | |  | |  | | | |  |
| 6 | At work, I have access to safe and clean water |  |  | |  | |  | |  | | | |  |
| 7 | At work, I have good access to electricity |  |  | |  | |  | |  | | | |  |
| 8 | I have efficient transportation to work |  |  | |  | |  | |  | | | |  |
| 9 | The community has good shopping areas and entertainment |  |  | |  | |  | |  | | | |  |
|  | **Statements of remuneration** | **Scale** |  | | | | | | | | |  |  |
|  |  | **Strongly disagree** | **Disagree** | **Neutral** | | **Agree** | | **Strongly agree** | | | | | |
| 1 | I feel I am being paid a fair amount for the work I do |  |  |  | |  | |  | | | | | |
| 2 | I am satisfied with the salary I receive |  |  |  | |  | |  | | | | | |
| 3 | I am provided with overtime/duty payment |  |  |  | |  | |  | | | | | |
| 4 | I feel the duty/overtime payment is sufficient and attractive |  |  |  | |  | |  | | | | | |
| 5 | I am provided with allowances for medical needs |  |  |  | |  | |  | | | | | |
| 6 | I am provided with allowances for housing facilities |  |  |  | |  | |  | | | | | |
| 7 | I am provided with risk covers and hardship allowances |  |  |  | |  | |  | | | | | |
| 8 | I feel there is sufficient opportunity for promotion |  |  |  | |  | |  | | | | | |
| 9 | Promotion in my organization is based on performance |  |  |  | |  | |  | | | | | |
| 10 | Promotion in my organization depends on the length of service |  |  |  | |  | |  | | | | | |
| 11 | I feel there is sufficient opportunity for continuing education |  |  |  | |  | |  | | | | | |
| 12 | I have been provided with on the job training needed to succeed in my position |  |  |  | |  | |  | | | | | |
|  | **Statements of supervision** | **Scale** |  |  |  |  |  |  |  |  |  |  |  |
|  |  | **Strongly disagree** | **Disagree** | **Neutral** | | **Agree** | | **Strongly agree** | | | | | |
| 1 | I receive recognition and appreciation for doing good work |  |  |  | |  | |  | | | | | |
| 2 | I am satisfied with the support and guidance of my supervisor |  |  |  | |  | |  | | | | | |
| 3 | My supervisor treats me/everybody fairly |  |  |  | |  | |  | | | | | |
| 4 | Selection of health workers for training is clear and transparent |  |  |  | |  | |  | | | | | |
| 5 | I feel the performance appraisal is not subjective |  |  |  | |  | |  | | | | | |
|  | **Statements of autonomy** | **Scale** |  |  |  |  |  |  |  |  |  |  |  |
|  |  | **Strongly disagree** | **Disagree** | **Neutral** | | **Agree** | | **Strongly agree** | | |  |  |  |
| 1 | I have sufficient authority to fulfill my job responsibilities |  |  |  | |  | |  | | |  |  |  |
| 2 | I am able to do my job independently of others |  |  |  | |  | |  | | |  |  |  |
| 3 | I have enough authority to make decisions necessary to provide quality treatment services |  |  |  | |  | |  | | |  |  |  |
|  | **Statements of peer group relationships** | **Scale** |  | | | | | | |  |  |  |  |
|  |  | **Strongly disagree** | **Disagree** | **Neutral** | | **Agree** | | **Strongly agree** | | |  |  |  |
| 1 | I have a very good relationship with my coworkers |  |  |  | |  | |  | | |  |  |  |
| 2 | I feel part of a team working for the good of our patients |  |  |  | |  | |  | | |  |  |  |
| 3 | There are people at work I can talk to when I need help |  |  |  | |  | |  | | |  |  |  |
| 4 | I am involved in the social network of the organization |  |  |  | |  | |  | | |  |  |  |

**Part 4: health professional’s organizational commitment questionnaire**

**Instruction: There are statements about health professional’s organizational commitment, and each state ment has five alternatives with five-point scale. Read each item carefully and tick:**

| **No** | **Statements of organizational commitment** | **Scale** | | | | |
| --- | --- | --- | --- | --- | --- | --- |
|  |  | **Strongly disagree** | **Disagree** | **Neutral** | **Agree** | **Strongly agree** |
| Affective commitment | | | | | | |
| 1 | I am quite proud to be able to tell people the organization for whom I work |  |  |  |  |  |
| 2 | I feel a sense of ownership for this organization rather than being just an employee |  |  |  |  |  |
| 3 | This organization has a great deal of personal meaning for me |  |  |  |  |  |
| 4 | I really feel as if this organizations problems are my own |  |  |  |  |  |
| Continuance commitment | | | | | | |
| 1 | It would be very hard for me to leave my organization right now even if I wanted to |  |  |  |  |  |
| 2 | I feel that I have too few options to consider leaving this organization(scarcity of available alternatives) |  |  |  |  |  |
| 3 | I stay in this organization because other organization cannot give me more than here |  |  |  |  |  |
| 4 | Too much of my life would be disrupted if I decide to leave the organization now. |  |  |  |  |  |
| Normative commitment | | | | | | |
| 1 | I do not feel any obligation to remain with my current organization |  |  |  |  |  |
|  |  |  |  |  |  |  |
| 2 | Even if it were to my advantage, I do not feel it would be right to leave my organization now |  |  |  |  |  |
| 3 | This organization deserves my loyalty |  |  |  |  |  |
